# Are Free Will Believers Nicer People? (Four Studies Suggest Not)

**DOI:** 10.1177/1948550618780732

**Published:** 2018-06-28

**Authors:** Damien L. Crone, Neil L. Levy

**Affiliations:** 1 The University of Melbourne, Melbourne, Victoria, Australia; 2 Macquarie University, Sydney, New South Wales, Australia; 3 University of Oxford, Oxford, Oxfordshire, United Kingdom

**Keywords:** agency, altruism, ethics/morality, helping/prosocial behavior, individual differences, morality, personality

## Abstract

Free will is widely considered a foundational component of Western moral and legal codes, and yet current conceptions of free will are widely thought to fit uncomfortably with much research in psychology and neuroscience. Recent research investigating the consequences of laypeople’s free will beliefs (FWBs) for everyday moral behavior suggests that stronger FWBs are associated with various desirable moral characteristics (e.g., greater helpfulness, less dishonesty). These findings have sparked concern regarding the potential for moral degeneration throughout society as science promotes a view of human behavior that is widely perceived to undermine the notion of free will. We report four studies (combined *N* = 921) originally concerned with possible mediators and/or moderators of the abovementioned associations. Unexpectedly, we found no association between FWBs and moral behavior. Our findings suggest that the FWB–moral behavior association (and accompanying concerns regarding decreases in FWBs causing moral degeneration) may be overstated.

All in all, it appears that belief in free will contributes to prosocial behavior. Virtuous actions that facilitate group harmony are promoted by belief in free will and undermined by deterministic beliefs. (Baumeister, Crescioni, & Alquist, 2010, p. 3)

It seems that when people stop believing they are free agents, they stop seeing themselves as blameworthy for their actions. Consequently, they act less responsibly and give in to their baser instincts. Vohs emphasized that this result is not limited to the contrived conditions of a lab experiment. “You see the same effects with people who naturally believe more or less in free will,” she said. (Cave, 2016)

Free will is widely considered a foundational component of Western moral and legal codes, and yet current conceptions of free will are widely thought to fit uncomfortably with much research in psychology and neuroscience (Glenn & Raine, 2013; Greene & Cohen, 2004; Wegner, 2004). Motivated by this apparent conflict, researchers have recently begun studying the consequences of laypeople’s free will beliefs (FWBs) for everyday moral behavior. The emerging picture is that FWBs have wide-ranging implications for moral behavior. Across experimental and correlational studies, stronger FWBs have been negatively associated with cheating and aggressive behavior (Baumeister, Masicampo, & Dewall, 2009; Vohs & Schooler, 2008) and positively associated with helpfulness, gratitude, job performance, and making amends for one’s transgressions (Baumeister et al., 2009; MacKenzie, Vohs, & Baumeister, 2014; Stillman & Baumeister, 2010; Stillman et al., 2010). As suggested by the opening quotes, these findings have sparked concern within, and outside of, academia regarding the potential for moral degeneration throughout society as science promotes a view of human behavior that is widely perceived to be at odds with the notion of free will (Baumeister et al., 2010; Cave, 2016; Shariff, Schooler, & Vohs, 2008; Shariff & Vohs, 2014). Such claims, if correct, would have wide-ranging implications across such areas as ethics, law, educational policy, and research funding and practice (e.g., should research with the potential to undermine folk notions of free will be funded and disseminated?). It is thus critical that our understanding of the association between FWBs and moral behavior rests on a solid evidence base.

The overwhelming majority of studies of the FWB–moral behavior association involve undermining FWBs and observing *momentary* lapses in moral behavior, with (to our knowledge) only one study testing the association between *dispositional* FWBs and moral behavior (Baumeister et al., 2009). As the opening quotes suggest, these findings have been collectively interpreted as implying that people with situationally *or dispositionally* low FWBs exhibit similar deficits in moral behavior. However, there is little data directly addressing the question of whether free will believers are generally nicer people. Here, we report four studies (combined *N* = 921) originally concerned with possible mediators and/or moderators of the FWB–moral behavior association. Unexpectedly, we found no association between FWBs and moral behavior.

## Method

Given the substantial overlap in methods across all four studies, we describe all four studies concurrently. All studies conducted for this project are reported, as are all experimental manipulations, and all measures pertinent to our central research question.^1^

### Power Analysis

To our knowledge, the only correlational study examining the relationship between dispositional belief in free will and prosocial or antisocial behavior is Study 2 of Baumeister, Masicampo, and Dewall (2009); all other studies of FWBs and moral behavior are experimental in nature (relying on undermining or boosting people’s FWBs rather than measuring preexisting beliefs). In the Baumeister et al. study, the authors observed a significant, positive association between FWBs and helping behavior (β = .30). Across all four studies, we achieved 80% power to detect correlations between .16 (Study 3) and .20 (Study 4), and in the pooled data analysis reported in the Supplementary Material (combining data from Studies 2 to 4), we had 80% power to detect a correlation of .10 (assuming any such effect is unrelated to the subtle methodological differences across studies).

### Participants

For all studies, participants were recruited via Amazon’s Mechanical Turk. Eligibility was restricted to workers located in the United States with approval rates ≥95%, and ≥1,000 previously approved jobs. A summary of demographic information for each study is provided in Table 1.

**Table 1.** Summary of Demographic Information.

Study	Final *N*	% Female	*M*_age_	*SD*_age_	% Christian	% FW
Study 1	210	46.19	35.63	12.72	40.48	—
Study 2	220	59.55	39.56	13.23	50.91	34.55
Study 3	294	51.70	37.89	12.24	48.64	35.37
Study 4	197	46.19	34.07	11.68	45.69	32.49

*Note.* % FW refers to percentage of participants who reported having previously participated in research on free will beliefs.

#### Exclusions

Participants were excluded if they either provided incomplete data or failed attention checks. In Studies 1–3, which included multiple attention checks, participants were excluded for failing more than one attention check. Because Study 4 had only one attention check, all participants failing this attention check were excluded. The number of people excluded per study is summarized in Table 2.

**Table 2.** Summary of Exclusions.

Study	Original *N*	Incomplete	Inattentive	Final *N*
Study 1	250	29	11	210
Study 2	243	22	1	220
Study 3	329	32	3	294
Study 4	288	89	2	197

### Materials and Procedure

The specific measures used in each study are summarized in Table 3. In all studies, we administered measures of FWBs, prosocial behavior, and moral identity. In Studies 2–4, we also included a measure of antisocial behavior. Additionally, Studies 2 and 3 included a measure of social desirability, and Study 4 included an unsuccessful FWB manipulation. These are both described further in the Supplementary Material.

**Table 3.** Summary of Key Measures.

		Prosocial Behavior		Antisocial Behavior	
Study	FWB (α)	Measure	Payoffs	Measure	Payoffs
1	FAD (.88), FWI (.88)	Dictator game	Self: US$0–US$0.10 Other: US$0–US$0.10 Max total: US$0.10	—	—
2	FAD (.86), FWI (.89)	SVO slider	Self: US$0.41–US$0.56 Other: US$0.23–US$0.52 Max total: US$0.90	Dice	US$0.01–US$0.06
3	FAD (.87), FWI (.85)	SVO slider	Self: US$51.75–US$72.75 Other: US$43–US$71 Max total: US$128.50	Dice	US$5–US$30
4	FAD (.90)^a^	SVO slider	Self: US$20.25–US$27.75 Other: US$11.50–US$26 Max total: US$45	Dice	US$5–US$30

*Note.* FWB = free will belief; FAD = FAD-Plus (Paulhus & Carey, 2011); FWI = Free Will Inventory (Nadelhoffer, Shepard, Nahmias, Sripada, & Ross, 2014); SVO Slider = social value orientation slider measure (Murphy, Ackermann, & Handgraaf, 2011).

^a^ Free Will subscale only.

#### FWB measures

In all four studies, participants completed the FAD-Plus (Paulhus & Carey, 2011). The FAD-Plus is a 27-item self-report measure of belief in free will in which participants rate the extent to which they agree with each statement on a scale from 1 (s*trongly disagree*) to 7 (*strongly agree*) with the statements forming four subscales: Free Will (e.g., “People have complete free will”), Scientific Determinism (e.g., “Your genes determine your future”), Fatalistic Determinism (e.g., “I believe that the future has already been determined by fate”), and Unpredictability (e.g., “People’s futures cannot be predicted”). In Study 4, participants only completed the Free Will subscale.

In Studies 1–3, participants also completed the Free Will Inventory (FWI; Nadelhoffer, Shepard, Nahmias, Sripada, & Ross, 2014). The FWI is a 29-item self-report measure of belief in free will, divided into two parts. In the first part (15 items), participants rated the extent to which they agree with each statement on a scale from 1 (*strongly disagree*) to 7 (*strongly agree*) with the statements forming three 5-item subscales: Free Will (e.g., “People always have the ability to do otherwise”), Determinism (e.g., “Given the way things were at the Big Bang, there is only one way for everything to happen in the universe after that”), and Dualism and Nonreductionism (e.g., “Each person has a nonphysical essence that makes that person unique”).

In the second part (14 items), using the same 7-point scale, participants report the extent of their agreement with individual statements regarding the relationships between free will, determinism, choice, the soul, predictability, responsibility, and punishment. As these items are not intended to form composite scales, we present analyses of each individual item in the Supplementary Material.

Both the FAD-Plus and FWI have undergone factor-analytic validation in multiple studies and samples (Nadelhoffer et al., 2014; Paulhus & Carey, 2011), and as shown in Table 3, both measures of FWBs were highly reliable (all αs ≥ .85 across all studies). Moreover, both measures were strongly correlated with each other (*r*s = .85, .82, and .76) across Studies 1–3.

#### Measures of prosocial behavior

In Study 1, participants completed a charity dictator game (DG). In the DG, participants were endowed with a 10-cent bonus (i.e., an additional 12.5% on top of their base payment) and given the opportunity to donate some or all of it to the American Red Cross.

For Study 2, we made multiple changes to our operationalization of prosocial behavior. First, given the substantial floor effect on generosity in Study 1 (48% of participants donated nothing), we sought to increase the value participants placed on the recipient by providing a choice between four different charities to nominate as the beneficiary in their allocation decisions, instead of having all donations directed to one charity.^2^

Second, instead of using a single DG allocation, we attempted to obtain a more sensitive measure of prosocial inclinations by using the social value orientation (SVO) slider measure (Murphy & Ackermann, 2014; Murphy, Ackermann, & Handgraaf, 2011). This measure entails a series of allocation decisions (much like mini DGs) that can be used to measure the weight one places on one’s own versus other’s interests.^3^ For each item, participants chose one of nine possible allocations with differing payoff structures. Participants distributed points between themselves and their nominated charity at an exchange rate that was described before participants began making their allocation decisions. These points were subsequently converted into money and paid out to both parties according to the participants’ decisions.

For Studies 3 and 4, we used the SVO slider measure again, but instead of allowing all participants to allocate a small amount of money, we increased the payoffs and instituted a lottery-based system where only a single randomly selected winner would have their allocations realized in each study. In Study 2 (where all participants were paid according to their choices), the exchange rate was 10 points to one cent. In Studies 3 and 4 (which employed lotteries), the exchange rate was one point to five cents (see Table 3 for further details). SVO angles were calculated using the MATLAB analysis scripts described in Murphy, Ackermann, and Handgraaf (2011).

#### Measures of antisocial behavior

To provide a complementary measure of moral behavior, Studies 2–4 also included a dice cheating task (Fischbacher & Föllmi-Heusi, 2013; Suri, Goldstein, & Mason, 2011), in which participants had the opportunity to lie without detection to earn a bonus. Specifically, participants were informed that they would roll a die, which would be used to determine the size of a bonus that they would receive. Participants were instructed to roll a die (either an actual one, or an online one, hosted on an independent website, whichever they preferred), privately record the number they rolled, and then proceed to the next page of the survey where they simultaneously (1) learned which numbers corresponded to which bonus size^4^ and (2) were given the opportunity to report the number they rolled. The fact that we could not observe any participant’s die roll made it possible for participants to lie to inflate the size of their bonus (e.g., rolling a one but reporting a five). Crucially, however, because of the known (i.e., uniform) distribution of dice rolls, it was possible to detect the presence of cheating at the group level and to link this to individual difference variables (e.g., to see whether people who disbelieve in free will are “luckier”). Similar to the measures of prosocial behavior, the payoffs in Study 2 were low but certain, and the payoffs in Studies 3 and 4 were substantially higher but realized for only one randomly selected participant per study (see Table 3 for further details).

#### Moral identity

Finally, in all four studies, we administered the Self-Importance of Moral Identity Questionnaire (SMI-Q; Aquino & Reed, 2002), which was included as a candidate moderator and also to affirm the validity of our outcome measures, given that the SMI-Q has been robustly associated with moral behavior (Hertz & Krettenauer, 2016). The SMI-Q is a 13-item self-report measure of the extent to which respondents view moral traits as central to their self. Participants rated the extent to which they agreed with each statement on a scale from 1 (s*trongly disagree*) to 7 (s*trongly agree*) with all statements being rated with regard to a hypothetical person with a set of nine traits (e.g., caring, compassionate, and fair). The scale consists of two subscales: the Internalization subscale (e.g., “It would make me feel good to be a person who has these characteristics”), which measures the centrality of moral traits to one’s self-concept, and the Symbolization subscale (e.g., “I often wear clothes that identify me as having these characteristics”), which measures the extent to which one behaves in ways that express these moral traits.

## Results

Given the highly similar structure of each study, we report results for all studies together. In this Results section, we present analyses of the association between FWBs and moral behavior in each individual study, as well as a meta-analytic summary of the four studies. Further information on the reliabilities, distributions, and correlations between key variables across studies is provided in the Supplementary Material.

**Figure 1.**
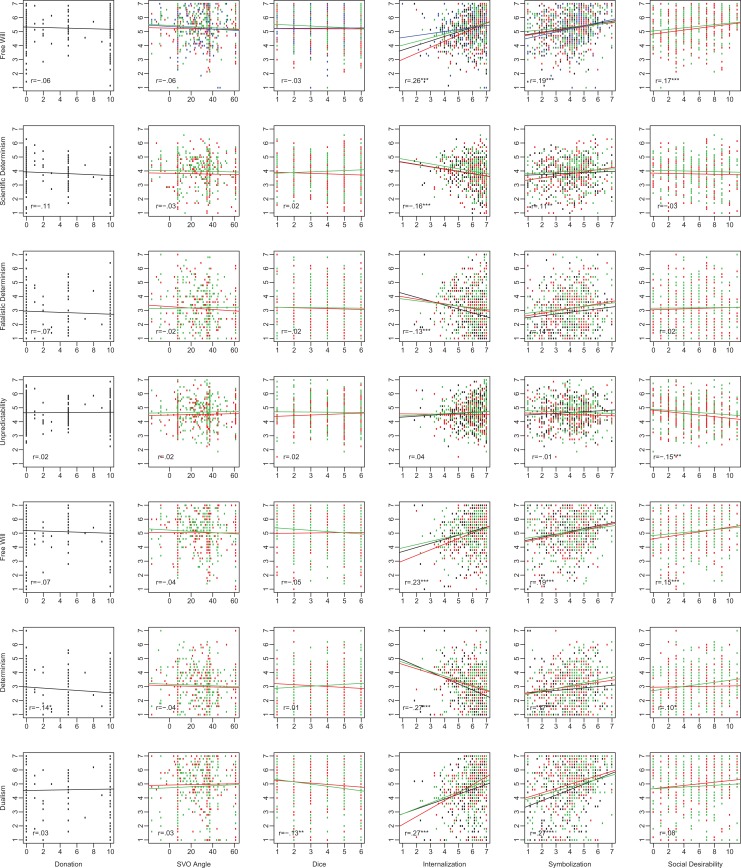
Bivariate distributions of free will beliefs (FWIs) and related belief measures (FAD-Plus subscales in Rows 1–4; FWI subscales in Rows 5–7) and measures of prosocial behavior (donation and social value orientation angle), antisocial behavior (dice), and moral identity and social desirability (columns). Point color represents study number (black = Study 1, red = Study 2, green = Study 3, and blue = Study 4). Where multiple studies are summarized in a single panel, correlation coefficients refer to combined data sets.

**Figure 2.**
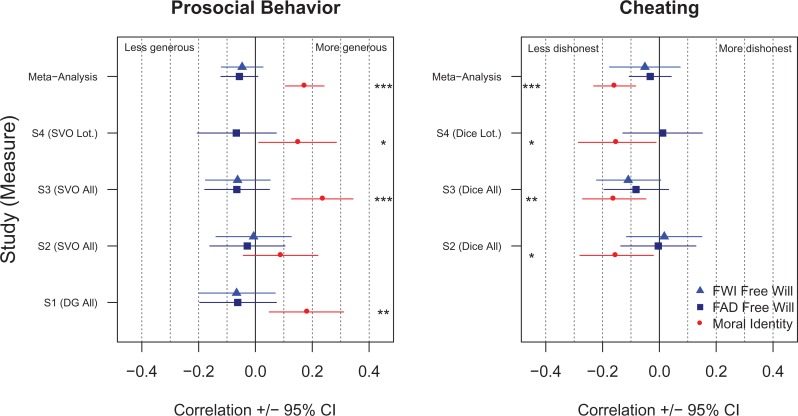
Correlation between free will beliefs and moral identity and prosocial behavior (left panel) and cheating behavior (right panel) across studies. DG = dictator game; SVO = social value orientation slider measure; Lot. = lottery; FWI = Free Will Inventory; FAD = FAD-Plus; moral identity refers to the Internalization subscale only; *p < .05. **p < .01. ***p < .001.

A summary of the correlations of primary interest across all four studies is presented in Figures 1 and 2, and a meta-analytic summary is presented in Table 4. In three of the four studies, moral identity was significantly, positively associated with generosity, with the meta-analytic correlation matching a recent meta-analytic estimate (*r* = .17) for observational measures of moral behavior (Hertz & Krettenauer, 2016). In all three studies containing measures of cheating behavior, moral identity was negatively associated with cheating. This pattern of findings demonstrates the validity of our outcome measures and that we had sufficient power to detect correlations with individual difference variables, even for our noisy, indirect measure of cheating.

**Table 4.** Summary of Meta-Analytic Effect Sizes and Heterogeneity Estimates.

Predictor	Generosity			Cheating		
	*r* (CI)	*p*	*I* ^2^ (CI)	*r* (CI)	*p*	*I* ^2^ (CI)
FAD-Plus						
Free will	−.06 [−.12, .01]	.088	0.00 [0.00, 2.33]	−.03 [−.11, .04]	.392	0.00 [0.00, 95.68]
Scientific determinism	−.05 [−.13, .02]	.164	0.00 [0.00, 95.81]	.02 [−.11, .14]	.806	53.41 [0.00, 99.95]
Fatalistic determinism	−.04 [−.11, .03]	.345	0.00 [0.00, 95.78]	.02 [−.11, .06]	.593	0.00 [0.00, 97.86]
Unpredictability	.03 [−.05, .10]	.490	0.00^a^	.02 [−.06, .12]	.597	7.63 [0.00, 99.91]
Free Will Inventory						
Free will	−.05 [−.12, .02]	.210	0.00 [0.00, 90.39]	−.05 [−.18, .07]	.421	50.26 [0.00, 99.95]
Determinism	−.06 [−.14, .001]	.077	0.00 [0.00, 97.09]	.01 [−.16, .17]	.937	70.45 [0.00, 99.97]
Dualism	.03 [−.04, .10]	.447	0.00 [0.00, 11.27]	−.13 [−.21, −.04]	**<.005**	0.00 [0.00, 99.80]
Moral identity						
Internalization	.17 [.11, .24]	**<.001**	8.45 [0.00, 92.15]	−.16 [−.23, −.08]	**<.001**	0.00^a^
Symbolization	.09 [.02, .15]	**<.010**	0.00 [0.00, 91.45]	−.07 [−.14, .01]	.077	0.00 [0.00, 96.82]

*Note.* Significant correlations (*p* < .05) in boldface. CI = confidence interval.

^a^ Heterogeneity estimate was negative; therefore, confidence intervals could not be computed.

Turning to FWBs, unexpectedly, across two measures of FWBs and all studies, we found no correlation with prosocial or antisocial behavior. In the case of prosocial behavior, the correlations were in fact uniformly negative: Stronger FWBs were (nonsignificantly) associated with *less* generosity. For all three measures of deterministic beliefs, we found no significant correlations with either moral behavior. Dualism was negatively correlated with cheating but unrelated to generosity.

### Meta-Analytic Summary

To summarize the correlations between FWBs and moral behavior, we computed 18 meta-analytic correlation coefficients (i.e., the meta-analytic correlation between subscales from the FAD-Plus, FWI, and SMI-Q on the one hand and generosity and cheating behavior on the other). Random effects meta-analyses were performed in *R*, with the *metafor* package (Viechtbauer, 2010), using Fisher’s *r*-to-*z* transformation and restricted maximum likelihood estimation.

Across all 18 meta-analyses, moral identity internalization and symbolization were significantly positively correlated with generosity. In addition, FWB as measured by the FAD-Plus and determinism as measured by the FWI were marginally *negatively* correlated with generosity. For cheating behavior, only dualism (measured by the FWI) and moral identity internalization were significantly negatively correlated with cheating, while symbolization was marginally negatively correlated with cheating. No other correlations were significant.

Estimates of effect size heterogeneity (due to such factors as variations in task payoff structure) were typically low but were also estimated with a low degree of precision, with extremely wide confidence intervals (CIs) for most cases. This is unsurprising, given the small number of studies included in the meta-analysis.

### Exploratory Analyses

Given our original aims, we conducted a series of exploratory analyses using aggregated data across Studies 2–4, to probe this surprising pattern of results (summarized in the Supplementary Material). These analyses provide tentative evidence for a range of suppression and interaction effects, which may provide fruitful leads for future investigations. Importantly though, none of these effects produced the expected salutary main effect of FWBs suggested by previous research.

## Discussion

The prevailing view in the behavioral sciences cautions that decreases in FWBs may be accompanied by deteriorating moral behavior (Cave, 2016; Shariff et al., 2008). Across four highly powered cross-sectional studies, we found no evidence to support such concerns: FWBs were neither clearly associated with increased generosity nor reduced cheating.

### Limitations

#### Was our cheating measure sufficiently sensitive?

One obvious limitation that may have hampered our ability to detect associations between FWBs and antisocial behavior is the relative imprecision of our outcome measure. By asking participants to report a single, unobserved dice roll, we introduced a substantial amount of noise into our measure of cheating.^5^ However, despite this imprecision, we were still able to infer the presence of cheating and still achieved sufficient power to consistently detect effects in the expected direction for moral identity internalization. Moreover, the meta-analytic point estimate of the correlation between moral identity and cheating (*r* = −.16) was well outside of the CIs for the association between FAD-Plus Free Will and cheating (95% CI [−.11, .04]), and the CIs for the two estimates overlapped only slightly. This suggests that, if FWBs are in fact negatively related to cheating, the association is likely to be trivially small in comparison to the association between cheating and more proximal variables such as one’s moral identity.

### Generalizability Across Dimensions of Belief and Kinds of Moral Behavior

Across all studies, our measures of prosocial and antisocial behavior were quite homogeneous. It is thus important to consider the extent to which our findings might hold for other operationalizations of moral behavior (although given the absence of any clear effects, this is quite a speculative exercise). Among the mechanisms proposed by Schooler, Nadelhoffer, Nahmias, and Vohs (2014) to explain the relationship between FWBs and antisocial behavior is the “exoneration account” in which undermining FWBs arms people with an excuse that can be deployed to explain their own misbehavior (or in the case of generosity, their lack of good behavior).^6^ Our results provide no support for this account. Instead, our results suggest that if FWBs are associated with moral behavior (and if such associations are explained by the exoneration account), the FWB–moral behavior association may only be limited to *specific kinds* of moral behavior. In contexts where cheating is clearly undetectable (such as our dice cheating task), being able to justify one’s own behavior may be a less important determinant of misbehavior. However, in other contexts where people could conceivably have to justify their behavior, we cannot rule out the possibility of a FWB–moral behavior association, and such an association may be well explained by the exoneration account. Similarly, for prosocial behavior, the exoneration account might predict that the effect of FWBs would emerge in less anonymous settings (e.g., interpersonal interactions or behavior observed by others), where people may feel a greater pressure to be able to justify their actions.

More generally, we note that (setting aside the lack of statistical significance) our meta-analytic results suggest that, if FWBs are associated with moral behavior, the pattern of associations is complex: Both measures of FWB were negatively associated with generosity but so too were all three measures of deterministic beliefs. In short, any potential association between FWBs and moral behavior is likely to be much smaller and/or more context-sensitive than previously suggested and potentially driven by multiple dimensions of FWBs.

## Conclusion

Across four studies, we found no evidence for a positive association between FWBs and desirable moral behavior. Considered in combination with (1) an independent, highly powered experiment that found no effect of an FWB manipulation on moral behavior (Monroe, Brady, & Malle, 2016, Study 1), (2) a study that only conceptually replicated the adverse effects of free will disbelief under very limited circumstances (among a small sample of nonreligious participants; Harms, Liket, Protzko, & Schölmerich, 2017), and (3) findings that seemingly contradict the notion that inducing free will disbelief (or related beliefs) produces antisocial behavior (Caspar, Vuillaume, Magalhaes De Saldanha Da Gama, & Cleeremans, 2017; Ma-Kellams & Blascovich, 2013), our findings suggest that the association between FWBs and moral behavior may be greatly overstated, with effects being smaller than previously reported or confined to specific contexts, subpopulations, or behaviors. As a result, we believe that there is good reason to doubt that FWBs have any substantial implications for everyday moral behavior. More research is required before actively discouraging free-will skepticism out of fear of moral degeneration (Cave, 2016; Vohs & Schooler, 2008).

## Supplemental Material

Supplemental Material, SPPS780732_suppl_mat - Are Free Will Believers Nicer People? (Four Studies Suggest Not) Click here for additional data file. Supplemental Material, SPPS780732_suppl_mat for Are Free Will Believers Nicer People? (Four Studies Suggest Not) by Damien L. Crone and Neil L. Levy in Social Psychological and Personality Science

## Notes

1. Other measures relating to separate research questions were also collected in these studies—these are to be reported elsewhere.

2. The four charities were The American Red Cross, Médecins Sans Frontières, The Against Malaria Foundation, and Habitat for Humanity.

3. Studies 2 and 4 used the six primary items from the Slider Measure. Study 3 also included the 9 secondary items designed to distinguish between two kinds of prosociality: inequality aversion and joint maximization (Murphy et al., 2011).

4. Payoffs increased linearly with outcomes, such that rolling a six paid 6 times as much as rolling a one.

5. The cheating measure could have been made more precise by using multiple trials, so that systematic misreporting becomes easier to detect at the group level, while still remaining unobserved at the individual level (Cohn, Fehr, & Maréchal, 2014; Hilbig & Thielmann, 2017; Purzycki et al., 2016). The use of multiple trials is thus an important methodological improvement that we encourage researchers to consider for future investigations.

6. The second explanation provided by Schooler et al. (2014) is based on ego depletion (Muraven & Baumeister, 2000), such that having one’s free will beliefs (FWBs) undermined taxes one’s self-control which, in turn, undermines moral behavior. We do not discuss this explanation in detail given that (1) there is uncertainty about whether ego depletion exists (Hagger et al., 2016), (2) Schooler et al. (2014) report only mixed support for the ego depletion account in their own work, and (3) although the ego depletion account may provide a plausible explanation for the effect of *manipulations* of FWBs, it is unclear how this explanation would apply to correlational studies (unless one assumed that those low in FWB were in a constant state of depletion).
