# Supplementary material for: Are Free Will Believers Nicer People? (Four Studies Suggest Not)
Source: Soc Psychol Personal Sci. 2018 Jun 28;10(5):612–9. doi: 10.1177/1948550618780732 (PMC6542011; doi:10.1177/1948550618780732)
Supplement: Supplemental Material, SPPS780732_suppl_mat - Are Free Will Believers Nicer People? (Four Studies Suggest Not) [file SPPS780732_suppl_mat.docx]

# Methods

## Additional materials

**Social desirability.** In addition to the measures reported in the main manuscript, in Studies 2 and 3, we administered a short form version of the Marlowe-Crowne Social Desirability Scale (MC-SDS; Crowne & Marlowe, 1960). Specifically, we used the 11-item version (Form A) developed by Reynolds (1982), and further validated by Loo and Thorpe (2000). The scale measures the tendency to give socially desirable responses by presenting a series of self-descriptive statements referring to desirable but very rare characteristics (e.g., “I am always courteous, even to people who are disagreeable”). Participants must rate each statement as either true or false. Across the Study 2 and 3, Cronbach’s alphas for the MC-SDS were .79 and .77, respectively.

**Free will beliefs manipulation.** In Study 4, we attempted a FWB manipulation to experimentally test the relation between FWBs and prosocial and antisocial behavior (however the manipulation was ultimately unsuccessful in shifting FWBs). The specific FWB manipulation used was adapted from Study 2 of Vohs and Schooler (2008), which was based on the Velten mood induction technique (Velten, 1968). In this task, participants were presented with ten statements (randomly drawn from a set of 15 statements) which they were required to view for a minimum of 30 seconds each, and re-write in their own words. Participants were randomly assigned to one of two conditions, with each condition containing a different set of statements. In the Anti-Free Will condition, participants were asked to rewrite statements intended to undermine their belief in free will (e.g., “Science has demonstrated that free will is an illusion”), whereas in the Control condition, participants were asked to rewrite factual statements with no relation to free will (e.g., “Oceans cover 71% of the earth's surface”).^[[1]](#footnote-1)^

# Results

In this supplementary results section, we present two sets of analyses: (1) a summary of the (unsuccessful) FWB manipulation in Study 4, and (2) a set of exploratory moderator analyses examining variability in the relationship between FWBs, and prosocial and antisocial behavior across Studies 2 through 4.

**Study 4 manipulation check.** To assess the success of our manipulation, we compared scores on the FAD-Plus Free Will subscale across the anti-free will and control conditions. Distributions of scores across the two conditions are shown in Figure 1. To test the success of the FWB manipulation conducted in Study 4, we performed a Welch two-sample t-test, comparing the average FWB endorsement of the anti-free will and control conditions. Although the group means were in the expected direction (anti-free will: *M* = 5.15, *SD* = 1.16; control: *M* = 5.34, *SD* = 1.11, see Figure 1), the effect of the manipulation on free will beliefs was non-significant (*t*(190.34) = 1.19, *p* = .24, mean difference 95% CI = -0.51, 0.13).

Figure 1. FAD-Plus Free Will endorsement across experimental conditions. Error bars represent +/- 2 SEs.

Given the unsuccessful manipulation, we examined whether the effectiveness of the manipulation was moderated by one (or both) of two factors: previous experience participating in research on free will, and participants’ religious beliefs. Regarding the first factor, given that all studies were conducted on AMT, it is possible that a non-trivial number of participants may have previously participated in similar studies (Chandler, Mueller, & Paolacci, 2014), which may have decreased the effectiveness of the manipulation.^[[2]](#footnote-2)^ Regarding religion, although some studies of North American, presumably Christian-dominant samples do report correlations between FWBs and religiosity (e.g., asking questions to the effect of “How religious are you?”) (Carey & Paulhus, 2013; Nadelhoffer, Shepard, Nahmias, Sripada, & Ross, 2014; Rakos, Laurene, Skala, & Slane, 2008), to our knowledge, no studies test for differences in FWBs between different religious groups (e.g., Christians vs. Atheists), or test for differences in the effectiveness of FWB manipulations as a function of religion. There is, however, a wealth of evidence for other related folk-psychological beliefs (e.g., about the extent to which one can control their thoughts or alter their fate) varying as a function of one’s religious or cultural background (Au et al., 2012; Cohen, 2003; Cohen & Rozin, 2001; Menon, Morris, Chiu, & Hong, 1999; Siev & Cohen, 2007). As such, it is plausible that a manipulation designed to undermine FWBs may have differentially affected people belonging to different religious groups.

To test for these effects, we performed four separate regressions, predicting our manipulation check measure (FAD-Plus Free Will) from (1) condition only (analogous to the t-test reported above), (2), condition, previous experience taking part in free will research, and their interaction, (3) condition, religion, and their interaction, and (4) all three predictors (with all interaction terms). These regression models are summarized in Table 1.

| Table 1  Moderation models predicting manipulation success from demographics | | | | | | | | | | | | |
| --- | --- | --- | --- | --- | --- | --- | --- | --- | --- | --- | --- | --- |
|  |  | Model 1 | |  | Model 2 | |  | Model 3 | |  | Model 4 | |
|  |  | *B (CI)* | *p* |  | *B (CI)* | *p* |  | *B (CI)* | *p* |  | *B (CI)* | *p* |
| (Intercept) |  | 5.34 (5.12 – 5.56) | **<.001** |  | 5.37 (5.11 – 5.63) | **<.001** |  | 5.06 (4.74 – 5.38) | **<.001** |  | 5.11 (4.70 – 5.51) | **<.001** |
| Anti-FW |  | -0.19 (-0.51 – 0.13) | .235 |  | 0.00 (-0.38 – 0.39) | .989 |  | -0.14 (-0.61 – 0.32) | .545 |  | 0.03 (-0.54 – 0.60) | .915 |
| Prev-FW |  |  |  |  | -0.09 (-0.55 – 0.37) | .701 |  |  |  |  | -0.13 (-0.79 – 0.53) | .693 |
| Anti-FW × Prev-FW |  |  |  |  | -0.61 (-1.28 – 0.06) | .076 |  |  |  |  | -0.59 (-1.56 – 0.39) | .235 |
| Rel-Christian |  |  |  |  |  |  |  | 0.60 (0.16 – 1.05) | **.009** |  | 0.57 (0.03 – 1.12) | **.040** |
| Rel-Other |  |  |  |  |  |  |  | 0.06 (-0.67 – 0.79) | .871 |  | -0.12 (-0.99 – 0.74) | .775 |
| Anti-FW × Rel-Christian |  |  |  |  |  |  |  | 0.02 (-0.63 – 0.67) | .946 |  | -0.07 (-0.85 – 0.71) | .863 |
| Anti-FW × Rel-Other |  |  |  |  |  |  |  | -0.56 (-1.64 – 0.51) | .304 |  | 0.06 (-1.37 – 1.49) | .936 |
| Prev-FW × Rel-Christian |  |  |  |  |  |  |  |  |  |  | 0.06 (-0.88 – 1.01) | .894 |
| Prev-FW × Rel-Other |  |  |  |  |  |  |  |  |  |  | 0.63 (-0.97 – 2.22) | .440 |
| Anti-FW × Prev-FW × Rel-Christian |  |  |  |  |  |  |  |  |  |  | 0.30 (-1.10 – 1.70) | .673 |
| Anti-FW × Prev-FW × Rel-Other |  |  |  |  |  |  |  |  |  |  | -1.09 (-3.36 – 1.17) | .343 |
| Observations |  | 197 | |  | 197 | |  | 197 | |  | 197 | |
| R^2^ / adj. R^2^ |  | .007 / .002 | |  | .047 / .032 | |  | .097 / .073 | |  | .135 / .084 | |
| F-statistics |  | 1.419 | |  | 3.177* | |  | 4.101** | |  | 2.625** | |
| Note: Anti-FW = Anti-Free Will condition; Prev-FW = Previous experience with free will studies. Reference category for religion is “None”. | | | | | | | | | | | | |

Across all four models, the only significant predictor of FWBs was a positive main effect of identifying as a Christian. Neither religion nor previous experience with free will research interacted with condition. As such, our analyses provide no indication that the failure of the manipulation was due to religion or previous research experience (the negative sign of the Anti-FW×Prev-FW coefficient actually suggests the manipulation was non-significantly *more* effective in experienced participants). Note however that, given the small number of participants in each cell (e.g., only 30 people in the Anti-Free Will condition had previously participated in free will research), these analyses are underpowered to detect all but the largest effects.

Given the failure of the manipulation to undermine participants’ FWBs, and given that the success of the manipulation was not moderated by any of the candidate moderators that we tested, we opted to treat the dataset as an additional replication dataset for Studies 1 through 3. We consider possible reasons for the failure of the manipulation in the supplementary Discussion section below.

## Exploratory moderation analyses

Given the similarity of the measurements in Studies 2 through 4, we pooled the datasets and performed a set of exploratory regressions. In particular, we tested whether (1) other beliefs relating to free will (e.g., deterministic or dualistic worldviews), (2) beliefs about the *meaning* of FW, or (3) a range of demographic variables either predicted or interacted with FWBs to predict prosocial or antisocial behavior. For all analyses, study number was included as a fixed effect, and all questionnaire predictors were mean-centered. Sample sizes for all models ranged from 514 (where data was available from just Studies 2 and 3) to 711 (where data was available from Studies 2 through 4). These models are summarized below in Table 2 through to Table 7 for prosocial behavior, and Table 8 to Table 13 for antisocial behavior. These are presented in the Appendix, given their size.

Because of the large number of models (and large number of significance tests), we limit ourselves to summarizing only statistically significant coefficients in models with significant F tests, while cautioning that these findings should be replicated before great confidence can be placed in them.^[[3]](#footnote-3)^ First and foremost, we note that across all models, FWBs were never associated with greater generosity or reduced cheating, either in isolation or in interaction with any other predictor.

**Demographics.** First, regarding demographic variables (see Table 2, Table 3, Table 8, and Table 9), we specified six separate models, predicting generosity and cheating from FWB along with main effects and interactions with FWB for (1) gender, (2) age, (3) income, (4) education, (5) previous experience participating in free will research, and (6) religion (including separate effects for religious group and the strength of one’s identification with their religious group).^[[4]](#footnote-4)^ Across these models, we found that females and older participants tended to be more generous, while Christian participants exhibited less cheating (in comparison to non-religious participants).^[[5]](#footnote-5)^

**Moral identity and social desirability.** Next, regarding moral identity and social desirability (see Table 4 and Table 10), we fitted three models, each predicting generosity and cheating from FWBs and (1) Moral Identity Internalization, (2) Moral Identity Symbolization, and (3) Social Desirability. Across these models, Internalization predicted both higher generosity and less cheating, consistent with a recent meta-analysis (Hertz & Krettenauer, 2016). Most interestingly, the generosity model including Internalization revealed an apparent suppression effect, such that the inclusion of Internalization produced a *negative* association between FWBs and generosity. That is, at a given level of moral identity internalization, stronger FWBs were associated with less generosity.

**Free will beliefs and related beliefs.** Finally, for free will-related beliefs (see Table 5 through Table 7 and Table 11 through Table 13), we fitted 19 models each predicting generosity and cheating from FWBs and additional FAD-Plus subscales (models 1-3), additional FWI subscales (models 4-5), and FWI supplementary items (models 6-19).

For general free will-related beliefs, we observed no significant main effects or interactions for any of the FAD-Plus or FWI subscales, for either generosity or cheating behavior. Turning to the supplementary items of the FWI, we explored whether generosity or cheating behavior related to participants’ beliefs regarding the *relationship between* free will, determinism, choice, the soul, predictability, responsibility, and punishment (either in isolation, or in interaction with self-reported strength of FWB). These models are summarized in Table 7 and Table 13. For models predicting generosity (Table 7), we observed positive effects of three items.^[[6]](#footnote-6)^ Specifically, participants tended to be more generous when they (a) believed that free will and determinism are compatible (Item 1), (b) endorsed the classical compatibilist view that free will is the capacity to act in accordance with one’s desires (Item 3), and (c) believed that moral responsibility is compatible with determinism (Item 12). For models predicting cheating behavior (Table 13), we did not observe any significant main effects of the supplementary items.

# Discussion

## Why was the manipulation unsuccessful?

There are multiple factors that could have contributed to the failure of our manipulation. Recent research suggests that there are many methodological intricacies that affect the success of FWB manipulations (Schooler, Nadelhoffer, Nahmias, & Vohs, 2014). As already noted, our manipulation differed from previous studies. To provide just two examples, to ensure participant engagement, we required participants to view statements for 30 seconds and then re-write them in their own words, whereas in Vohs and Schooler (2008, Study 2), participants completed the study in person, and viewed statements for one minute, but only passively read them. We are unsure of which (if any) specific methodological difference (or combination of differences) undermined the manipulation,^[[7]](#footnote-7)^ but other similarly designed and well-powered studies have successfully used a variant of the Velten-type manipulation of FWBs on AMT (Monroe, Brady, & Malle, 2016).

Design issues aside, statistical power may have also contributed to our null findings. The study most similar to our own (Monroe et al., 2016, Study 1; published subsequent to us completing all data collection), used a Velten-type manipulation on AMT, and used FWI subscales as manipulation check measures, producing a manipulation check effect size of *d* = 0.21 for the FWI Free Will subscale.^[[8]](#footnote-8)^ A power analysis based on this Monroe et al. effect size suggests that, given our sample size in Study 4, we had only around 55% power to observe a significant effect for our manipulation check. Thus overall, we cannot be certain whether the failure of the manipulation was design-driven, or an issue of statistical power, or both.^[[9]](#footnote-9)^

# References

Au, E. W. M., Chiu, C., Zhang, Z.-X., Mallorie, L., Chaturvedi, A., Viswanathan, M., & Savani, K. (2012). Negotiable fate: Social ecological foundation and psychological functions. *Journal of Cross-Cultural Psychology*, *43*(6), 931–942. http://doi.org/10.1177/0022022111421632

Carey, J. M., & Paulhus, D. L. (2013). Worldview implications of believing in free will and/or determinism: Politics, morality, and punitiveness. *Journal of Personality*, *81*(2), 130–141. http://doi.org/10.1111/j.1467-6494.2012.00799.x

Chandler, J. J., Mueller, P., & Paolacci, G. (2014). Nonnaïveté among Amazon Mechanical Turk workers: consequences and solutions for behavioral researchers. *Behavior Research Methods*, *46*(1), 112–30. http://doi.org/10.3758/s13428-013-0365-7

Cohen, A. B. (2003). Religion, likelihood of action, and the morality of mentality. *International Journal for the Psychology of Religion*, *13*(4), 273–285. http://doi.org/10.1207/S15327582IJPR1304_4

Cohen, A. B., & Rozin, P. (2001). Religion and the morality of mentality. *Journal of Personality and Social Psychology*, *81*(4), 697–710. http://doi.org/10.1037//0022-3514.81.4.697

Crowne, D. P., & Marlowe, D. (1960). A new scale of social desirability independent of psychopathology. *Journal of Consulting Psychology*, *24*(4), 349–354. http://doi.org/10.1037/h0047358

Galen, L. W. (2012). Does religious belief promote prosociality? A critical examination. *Psychological Bulletin*, *138*(5), 876–906. http://doi.org/10.1037/a0028251

Hertz, S. G., & Krettenauer, T. (2016). Does moral identity effectively predict moral behavior?: A meta-analysis. *Review of General Psychology*, *20*(2), 129–140. http://doi.org/10.1037/gpr0000062

Loo, R., & Thorpe, K. (2000). Confirmatory factor analyses of the full and short versions of the Marlowe-Crowne Social Desirability Scale. *The Journal of Social Psychology*, *140*(5), 628–35. http://doi.org/10.1080/00224540009600503

Menon, T., Morris, M. W., Chiu, C., & Hong, Y. (1999). Culture and the construal of agency: Attribution to individual versus group dispositions. *Journal of Personality and Social Psychology*, *76*(5), 701–717. http://doi.org/10.1037/0022-3514.76.5.701

Monroe, A. E., Brady, G., & Malle, B. F. (2016). This isn’t the free will worth looking for: General free will beliefs do not influence moral judgments, agent-specific choice ascriptions do. *Social Psychological and Personality Science*, 1948550616667616. http://doi.org/10.1177/1948550616667616

Murphy, R. O., Ackermann, K. A., & Handgraaf, M. J. J. (2011). Measuring Social Value Orientation. *Judgment and Decision Making*, *6*(8), 771–781.

Murray, D., & Nahmias, E. (2014). Explaining Away Incompatibilist Intuitions. *Philosophy and Phenomenological Research*, *88*(2), 434–467. http://doi.org/10.1111/j.1933-1592.2012.00609.x

Nadelhoffer, T., Shepard, J., Nahmias, E., Sripada, C. S., & Ross, L. T. (2014). The Free Will Inventory: Measuring beliefs about agency and responsibility. *Consciousness and Cognition*, *25*(1), 27–41. http://doi.org/10.1016/j.concog.2014.01.006

Nahmias, E., Morris, S. G., Nadelhoffer, T., & Turner, J. (2005). Surveying freedom: Folk Intuitions about free will and moral responsibility. *Philosophical Psychology*, *18*(5), 561–584. http://doi.org/10.1080/09515080500264180

Nahmias, E., Morris, S. G., Nadelhoffer, T., & Turner, J. (2006). Is incompatibilism intuitive? *Philosophy and Phenomenological Research*, *73*(1), 28–53. http://doi.org/10.1111/j.1933-1592.2006.tb00603.x

Paulhus, D. L., & Carey, J. M. (2011). The FAD-Plus: Measuring lay beliefs regarding free will and related constructs. *Journal of Personality Assessment*, *93*(1), 96–104. http://doi.org/10.1080/00223891.2010.528483

Rakos, R. F., Laurene, K. R., Skala, S., & Slane, S. (2008). Belief in free will: Measurement and conceptualization innovations. *Behavior and Social Issues*, *17*, 20–39.

Reynolds, W. M. (1982). Development of reliable and valid short forms of the Marlowe-Crowne Social Desirability Scale. *Journal of Clinical Psychology*, *38*(1972), 119–126. http://doi.org/10.1002/1097-4679(198201)38:1<119::AID-JCLP2270380118>3.0.CO;2-I

Schooler, J. W., Nadelhoffer, T., Nahmias, E., & Vohs, K. D. (2014). Measuring and manipulating beliefs and behaviors associated with free will: The good, the bad, and the ugly. In A. R. Mele (Ed.), *Surrounding Free Will: Philosophy, Psychology, Neuroscience* (pp. 72–94). http://doi.org/10.1093/acprof:oso/9780199333950.001.0001

Siev, J., & Cohen, A. B. (2007). Is thought–action fusion related to religiosity? Differences between Christians and Jews. *Behaviour Research and Therapy*, *45*(4), 829–837. http://doi.org/10.1016/j.brat.2006.05.001

Suri, S., Goldstein, D. G., & Mason, W. A. (2011). Honesty in an online labor market. In *Workshops at the Twenty-Fifth AAAI Conference on Artificial Intelligence* (pp. 61–66). San Francisco, CA. Retrieved from http://www.aaai.org/ocs/index.php/WS/AAAIW11/paper/download/3955/4262

Velten, E. (1968). A laboratory task for induction of mood states. *Behaviour Research and Therapy*, *6*(4), 473–482. http://doi.org/10.1016/0005-7967(68)90028-4

Vohs, K. D., & Schooler, J. W. (2008). The value of believing in free will: Encouraging a belief in determinism increases cheating. *Psychological Science*, *19*(1), 49–54. http://doi.org/10.1111/j.1467-9280.2008.02045.x

# Appendix

## Distribution of free will beliefs

Univariate and bivariate distributions for FWB and moral identity measures are separately presented in Figure 2 and Figure 3. Across both measures, we reproduced two commonly observed phenomena. First, we observed a substantial negative skew in FWB, such that most participants were at or near the ceiling of the scale (Paulhus & Carey, 2011). Second, we observed a small-to-non-existent negative correlation between FWB and deterministic beliefs, providing further support for folk compatibilism (i.e., that people often endorse FWBs and deterministic beliefs simultaneously) (Murray & Nahmias, 2014; Nadelhoffer et al., 2014; Nahmias, Morris, Nadelhoffer, & Turner, 2005, 2006; Paulhus & Carey, 2011). It is important to note however, that unlike FWBs, few participants *strongly* endorsed deterministic beliefs across any of the measures.

Figure 2. Distribution of free will beliefs across studies, as measured by the FAD-Plus. Point color represents study number (Black = Study 1; Red = Study 2; Green = Study 3; Blue = Study 4). On diagonal: density plots for individual studies (thin grey lines) and all studies combined (thick black line), with Cronbach’s alphas for each study inset. Error bars on diagonal represent standard deviations.

Figure 3. Distribution of free will beliefs across studies, as measured by the FWI. Point color represents study number (Black = Study 1; Red = Study 2; Green = Study 3). On diagonal: density plots for individual studies (thin grey lines) and all studies combined (thick black line), with Cronbach’s alphas for each study inset. Error bars on diagonal represent standard deviations.

## Distribution of moral identity and moral behavior

Distributions of moral identity, and prosocial and antisocial behavior measures across studies are summarized in Figure 4 through Figure 6.

Figure 4. Distribution of Moral Identity across studies. Point color represents study number (Black = Study 1; Red = Study 2; Green = Study 3; Blue = Study 4). On diagonal: density plots for individual studies (thin grey lines) and all studies combined (thick black line), with Cronbach’s alphas for each study inset. Error bars on diagonal represent standard deviations.

Similar to the measures of FWBs, Moral Identity Internalization was negatively skewed such that most participants scored at or near the ceiling of the scale (see Figure 4). Far more variability was observed for the Symbolization subscale.

Figure 5. Distribution of prosocial behavior measures across studies. Note: Blue bars represent mean +/- 1 standard deviation; Letters (and numbers in parentheses) represent the number of people categorized as Competitive (C), Individualistic (I), Prosocial (P), and Altruistic (A); Dashed vertical lines denote angle thresholds for each SVO category.

For our behavioral measures of prosocial behavior (Figure 5), participants exhibited substantial variability in their generosity. For the charity dictator game (Study 1), around half of participants donated nothing, while around 30% donated their entire bonus, with others donating some amount in-between. For the SVO Slider Measure (Studies 2 through 4), participants were predominantly Individualistic (tending to either maximize their own payoffs without regard for their nominated charity’s payoffs) or Prosocial (tending to maximize joint payoffs, or minimize payoff discrepancies) according to the cutoffs described in Murphy et al. (2011). A small number of participants in each study were classified as Competitive (tending to maximize their own relative payoffs) or altruists (tending to maximize their nominated charity’s relative payoffs).

Figure 6. Distribution of dice rolls across Studies 2 through 4. Outcome-wise significance tests denote binomial tests separately comparing the frequency of each outcome to chance (i.e., $\frac{1}{6}$). Kolmogorov-Smirnov (KS) test results comparing observed distribution to a uniform distribution (i.e., an equal occurrence of each discrete outcome) inset.

Finally, for our measure of antisocial behavior, summarized in Figure 6, outcomes from the dice-rolling task showed clear, systematic departures from what would be expected from chance. To determine whether cheating occurred, we conducted two sets of analyses. The first set of tests entailed separate Kolmogorov-Smirnov tests (KS-test) for Studies 2 through 4, comparing the distribution of dice rolls to chance (i.e., to a uniform distribution where one sixth of participants report each outcome, representing a null hypothesis of accurate reporting of dice rolls). We observed significant departures from a uniform distribution in two of three studies, with marginal results in the third case.^[[10]](#footnote-10)^

The second set of analyses entailed a series of binomial tests that aimed to describe how participants’ dice rolls departed from the expected distribution (i.e., which outcomes were over- or under-reported). Specifically, we performed 18 separate binomial tests for each outcome in each study (i.e., separately comparing the expected to observed frequency for the six potential outcomes across three studies). The results of these tests are summarized in Figure 6. In all three studies, the lowest-paying outcome (i.e., rolling a one) was significantly under-reported, and one or both of the highest-paying outcomes (i.e., a five or six) were significantly over-reported. Overall, these analyses clearly suggest the presence of cheating, consistent with previous research (Suri, Goldstein, & Mason, 2011).

## Exploratory moderation analyses tables

Exploratory moderation models predicting generosity and cheating across Studies 2 through 4 are summarized below in Table 2 through Table 13.

| Table 2  Moderation models predicting SVO Angle from FAD Free Will, gender, age and income | | | | | | | | | | | | |
| --- | --- | --- | --- | --- | --- | --- | --- | --- | --- | --- | --- | --- |
|  |  | Baseline | |  | Gender | |  | Age | |  | Income | |
|  |  | *B (CI)* | *p* |  | *B (CI)* | *p* |  | *B (CI)* | *p* |  | *B (CI)* | *p* |
| (Intercept) |  | 27.52 (25.22 – 29.81) | **<.001** |  | 24.30 (21.57 – 27.03) | **<.001** |  | 21.47 (16.81 – 26.14) | **<.001** |  | 24.19 (20.43 – 27.95) | **<.001** |
| Study 3 |  | -1.98 (-5.02 – 1.05) | .200 |  | -1.54 (-4.55 – 1.47) | .315 |  | -1.62 (-4.65 – 1.40) | .292 |  | -1.99 (-5.04 – 1.07) | .201 |
| Study 4 |  | -1.72 (-5.06 – 1.61) | .310 |  | -1.02 (-4.34 – 2.29) | .545 |  | -0.74 (-4.11 – 2.63) | .668 |  | -1.50 (-4.85 – 1.85) | .380 |
| FW |  | -0.84 (-2.00 – 0.32) | .154 |  | -1.33 (-3.10 – 0.45) | .143 |  | 1.22 (-2.31 – 4.74) | .499 |  | -1.22 (-3.86 – 1.42) | .364 |
| Female |  |  |  |  | 5.36 (2.81 – 7.90) | **<.001** |  |  |  |  |  |  |
| FW × Female |  |  |  |  | 0.59 (-1.74 – 2.92) | .617 |  |  |  |  |  |  |
| Age |  |  |  |  |  |  |  | 0.15 (0.05 – 0.26) | **.004** |  |  |  |
| FW × Age |  |  |  |  |  |  |  | -0.06 (-0.15 – 0.03) | .191 |  |  |  |
| Income ($20k - $39k) |  |  |  |  |  |  |  |  |  |  | 4.25 (0.24 – 8.26) | **.038** |
| Income ($40k - $69k) |  |  |  |  |  |  |  |  |  |  | 2.32 (-1.94 – 6.58) | .285 |
| Income ($60k - $79k) |  |  |  |  |  |  |  |  |  |  | 4.48 (-0.19 – 9.15) | .060 |
| Income ($80k+) |  |  |  |  |  |  |  |  |  |  | 4.72 (0.33 – 9.12) | **.035** |
| FW × Income ($20k - $39k) |  |  |  |  |  |  |  |  |  |  | 0.18 (-3.33 – 3.69) | .920 |
| FW × Income ($40k - $69k) |  |  |  |  |  |  |  |  |  |  | 1.15 (-2.46 – 4.77) | .532 |
| FW × Income ($60k - $79k) |  |  |  |  |  |  |  |  |  |  | -0.81 (-4.85 – 3.23) | .694 |
| FW × Income ($80k+) |  |  |  |  |  |  |  |  |  |  | 0.36 (-3.51 – 4.24) | .855 |
| Observations |  | 711 | |  | 711 | |  | 711 | |  | 711 | |
| R^2^ / adj. R^2^ |  | .006 / .001 | |  | .030 / .023 | |  | .020 / .013 | |  | .016 / .001 | |
| F-statistics |  | 1.347 | |  | 4.287*** | |  | 2.852* | |  | 1.034 | |
| Note: Free will belief scales have been standardized; FW = FAD Free Will subscale | | | | | | | | | | | | |

| Table 3  Moderation models predicting SVO Angle from FAD Free Will, education, non-naïveté, and religion | | | | | | | | | |
| --- | --- | --- | --- | --- | --- | --- | --- | --- | --- |
|  |  | Education | |  | Prev. FW | |  | Religion | |
|  |  | *B (CI)* | *p* |  | *B (CI)* | *p* |  | *B (CI)* | *p* |
| (Intercept) |  | 27.61 (25.23 – 29.99) | **<.001** |  | 26.95 (24.48 – 29.43) | **<.001** |  | 25.88 (22.96 – 28.80) | **<.001** |
| Study 3 |  | -2.01 (-5.05 – 1.03) | .194 |  | -1.92 (-4.96 – 1.12) | .215 |  | -1.32 (-4.39 – 1.76) | .401 |
| Study 4 |  | -1.73 (-5.08 – 1.61) | .309 |  | -1.76 (-5.09 – 1.58) | .302 |  | -1.41 (-4.75 – 1.93) | .408 |
| FW |  | -1.05 (-2.31 – 0.22) | .104 |  | -0.42 (-1.82 – 0.97) | .552 |  | -1.30 (-3.22 – 0.61) | .182 |
| HS |  | -0.95 (-4.76 – 2.86) | .625 |  |  |  |  |  |  |
| FW × HS |  | 1.50 (-1.78 – 4.78) | .369 |  |  |  |  |  |  |
| Prev. FW |  |  |  |  | 1.50 (-1.18 – 4.19) | .272 |  |  |  |
| FW × Prev. FW |  |  |  |  | -1.26 (-3.77 – 1.26) | .326 |  |  |  |
| Christian |  |  |  |  |  |  |  | 2.03 (-0.86 – 4.93) | .168 |
| Other rel. |  |  |  |  |  |  |  | 3.42 (-1.28 – 8.12) | .154 |
| Rel. Str. |  |  |  |  |  |  |  | 0.72 (0.09 – 1.36) | **.026** |
| FW × Christian |  |  |  |  |  |  |  | 0.26 (-2.51 – 3.02) | .856 |
| FW × Other rel. |  |  |  |  |  |  |  | -0.72 (-4.43 – 3.00) | .704 |
| FW × Rel. Str. |  |  |  |  |  |  |  | 0.36 (-0.20 – 0.93) | .209 |
| Christian × Rel. Str. |  |  |  |  |  |  |  | -0.55 (-1.53 – 0.44) | .275 |
| Other × Rel. Str. |  |  |  |  |  |  |  | -0.70 (-2.35 – 0.95) | .403 |
| FW × Christian × Rel. Str. |  |  |  |  |  |  |  | 0.14 (-0.79 – 1.07) | .766 |
| FW × Other × Rel. Str. |  |  |  |  |  |  |  | -0.52 (-1.86 – 0.81) | .443 |
| Observations |  | 711 | |  | 711 | |  | 705 | |
| R^2^ / adj. R^2^ |  | .007 / -.000 | |  | .009 / .002 | |  | .029 / .010 | |
| F-statistics |  | 0.992 | |  | 1.258 | |  | 1.567 | |
| Note: Free will belief scales have been standardized; FW = FAD Free Will subscale; HS = High-school is highest level of education completed; Prev. FW = Previous experience with free will research; Rel. Str. = strength of identification with one’s religious group (standardized) | | | | | | | | | |

| Table 4  Moderation models predicting SVO Angle from moral identity and social desirability | | | | | | | | | |
| --- | --- | --- | --- | --- | --- | --- | --- | --- | --- |
|  |  | Internalization | |  | Symbolization | |  | Social Desirability | |
|  |  | *B (CI)* | *p* |  | *B (CI)* | *p* |  | *B (CI)* | *p* |
| (Intercept) |  | 27.00 (24.68 – 29.31) | **<.001** |  | 27.72 (25.42 – 30.03) | **<.001** |  | 27.54 (25.19 – 29.89) | **<.001** |
| Study 3 |  | -1.69 (-4.68 – 1.31) | .269 |  | -2.19 (-5.22 – 0.85) | .157 |  | -1.94 (-5.03 – 1.14) | .217 |
| Study 4 |  | -0.61 (-3.93 – 2.71) | .719 |  | -2.05 (-5.39 – 1.28) | .227 |  |  |  |
| FW |  | -1.64 (-2.82 – -0.45) | **.007** |  | -1.11 (-2.29 – 0.07) | .065 |  | -1.07 (-2.49 – 0.36) | .142 |
| Internalization |  | 3.25 (1.97 – 4.53) | **<.001** |  |  |  |  |  |  |
| FW × Internalization |  | 0.31 (-0.77 – 1.38) | .573 |  |  |  |  |  |  |
| Symbolization |  |  |  |  | 1.20 (0.23 – 2.18) | **.015** |  |  |  |
| FW × Symbolization |  |  |  |  | -0.11 (-0.96 – 0.73) | .793 |  |  |  |
| Soc. Des |  |  |  |  |  |  |  | 0.63 (0.10 – 1.16) | **.020** |
| FW × Soc. Des |  |  |  |  |  |  |  | -0.09 (-0.58 – 0.41) | .728 |
| Observations |  | 711 | |  | 711 | |  | 514 | |
| R^2^ / adj. R^2^ |  | .040 / .033 | |  | .014 / .007 | |  | .016 / .009 | |
| F-statistics |  | 5.853*** | |  | 2.001 | |  | 2.111 | |
| Note: Free will belief, moral identity, and social desirability scales have been standardized; FW = FAD Free Will subscale; Soc. Des. = Social Desirability | | | | | | | | | |

| Table 5  Moderation models predicting SVO Angle from free will beliefs as measured by the FAD Plus | | | | | | | | | | | | |
| --- | --- | --- | --- | --- | --- | --- | --- | --- | --- | --- | --- | --- |
|  |  | Baseline | |  | Scientific Determinism | |  | Fatalistic Determinism | |  | Unpredictability | |
|  |  | *B (CI)* | *p* |  | *B (CI)* | *p* |  | *B (CI)* | *p* |  | *B (CI)* | *p* |
| (Intercept) |  | 27.52 (25.18 – 29.86) | **<.001** |  | 27.46 (25.11 – 29.81) | **<.001** |  | 27.76 (25.42 – 30.10) | **<.001** |  | 27.56 (25.21 – 29.91) | **<.001** |
| Study 3 |  | -1.99 (-5.09 – 1.10) | .207 |  | -1.89 (-5.00 – 1.23) | .235 |  | -1.94 (-5.02 – 1.15) | .218 |  | -2.09 (-5.21 – 1.02) | .188 |
| FW |  | -0.79 (-2.20 – 0.62) | .273 |  | -0.81 (-2.23 – 0.61) | .261 |  | -0.89 (-2.31 – 0.53) | .217 |  | -0.75 (-2.16 – 0.67) | .301 |
| SD |  |  |  |  | -0.49 (-2.04 – 1.06) | .533 |  |  |  |  |  |  |
| FW × SD |  |  |  |  | -0.04 (-1.33 – 1.26) | .958 |  |  |  |  |  |  |
| FD |  |  |  |  |  |  |  | -0.73 (-1.92 – 0.45) | .224 |  |  |  |
| FW × FD |  |  |  |  |  |  |  | 1.29 (0.22 – 2.35) | **.018** |  |  |  |
| Unpred. |  |  |  |  |  |  |  |  |  |  | 0.48 (-1.16 – 2.12) | .566 |
| FW × Unpred. |  |  |  |  |  |  |  |  |  |  | -0.22 (-1.61 – 1.17) | .754 |
| Observations |  | 514 | |  | 514 | |  | 514 | |  | 514 | |
| R^2^ / adj. R^2^ |  | .006 / .002 | |  | .007 / -.001 | |  | .018 / .010 | |  | .007 / -.001 | |
| F-statistics |  | 1.496 | |  | 0.852 | |  | 2.278 | |  | 0.845 | |
| Note: Free will belief scales have been standardized; FW = Free Will subscale; SD = Scientific Determinism subscale; FD = Fatalistic Determinism subscale; Unpred. = Unpredictability subscale | | | | | | | | | | | | |

| Table 6  Moderation models predicting SVO Angle from free will beliefs as measured by the Free Will Inventory | | | | | | | | | |
| --- | --- | --- | --- | --- | --- | --- | --- | --- | --- |
|  |  | Baseline | |  | Determinism | |  | Dualism | |
|  |  | *B (CI)* | *p* |  | *B (CI)* | *p* |  | *B (CI)* | *p* |
| (Intercept) |  | 27.55 (25.22 – 29.89) | **<.001** |  | 27.28 (24.90 – 29.67) | **<.001** |  | 27.36 (24.98 – 29.74) | **<.001** |
| Study 3 |  | -2.06 (-5.15 – 1.03) | .191 |  | -1.77 (-4.89 – 1.36) | .267 |  | -1.93 (-5.03 – 1.18) | .223 |
| FW |  | -0.50 (-1.72 – 0.71) | .416 |  | -0.63 (-1.86 – 0.61) | .318 |  | -0.58 (-1.86 – 0.69) | .370 |
| Determinism |  |  |  |  | -0.57 (-1.79 – 0.66) | .366 |  |  |  |
| FW × Determinism |  |  |  |  | -0.46 (-1.33 – 0.41) | .298 |  |  |  |
| Dualism |  |  |  |  |  |  |  | 0.44 (-0.53 – 1.42) | .373 |
| FW × Dualism |  |  |  |  |  |  |  | 0.23 (-0.40 – 0.86) | .482 |
| Observations |  | 514 | |  | 514 | |  | 514 | |
| R^2^ / adj. R^2^ |  | .005 / .001 | |  | .009 / .001 | |  | .007 / -.001 | |
| F-statistics |  | 1.224 | |  | 1.098 | |  | 0.933 | |
| Note: Free will belief scales have been standardized; FW = Free Will subscale | | | | | | | | | |

| Table 7  Moderation models predicting SVO Angle from free will beliefs and supplementary items from the Free Will Inventory | | | | | | | | | | | | | | | | | | | | | | |
| --- | --- | --- | --- | --- | --- | --- | --- | --- | --- | --- | --- | --- | --- | --- | --- | --- | --- | --- | --- | --- | --- | --- |
|  |  | 1 | |  | 2 | |  | 3 | |  | 4 | |  | 5 | |  | 6 | |  | 7 | |  |
|  |  | *B (CI)* | *p* |  | *B (CI)* | *p* |  | *B (CI)* | *p* |  | *B (CI)* | *p* |  | *B (CI)* | *p* |  | *B (CI)* | *p* |  | *B (CI)* | *p* |  |
| (Intercept) |  | 27.26 (24.85 – 29.67) | **<.001** |  | 27.87 (25.49 – 30.25) | **<.001** |  | 27.18 (24.77 – 29.59) | **<.001** |  | 27.53 (25.18 – 29.89) | **<.001** |  | 27.20 (24.74 – 29.67) | **<.001** |  | 27.59 (25.21 – 29.97) | **<.001** |  | 27.06 (24.67 – 29.45) | **<.001** |  |
| Study 3 |  | -1.64 (-4.74 – 1.45) | .296 |  | -1.98 (-5.07 – 1.11) | .209 |  | -1.94 (-5.02 – 1.14) | .217 |  | -2.13 (-5.23 – 0.97) | .178 |  | -1.90 (-5.01 – 1.22) | .232 |  | -2.04 (-5.13 – 1.05) | .196 |  | -1.89 (-4.97 – 1.20) | .230 |  |
| FW |  | -1.21 (-2.51 – 0.09) | .069 |  | -0.71 (-2.00 – 0.58) | .282 |  | -1.16 (-2.48 – 0.16) | .084 |  | -0.57 (-1.85 – 0.71) | .384 |  | -0.62 (-2.05 – 0.81) | .394 |  | -0.09 (-1.42 – 1.24) | .893 |  | -0.87 (-2.18 – 0.45) | .196 |  |
| Item |  | 1.96 (0.63 – 3.29) | **.004** |  | 0.67 (-0.50 – 1.85) | .260 |  | 1.72 (0.43 – 3.01) | **.009** |  | 0.33 (-0.65 – 1.32) | .507 |  | 0.38 (-0.83 – 1.60) | .535 |  | -0.89 (-1.90 – 0.11) | .082 |  | 1.08 (-0.02 – 2.17) | .055 |  |
| FW × Item |  | 0.09 (-0.81 – 1.00) | .837 |  | -0.63 (-1.45 – 0.19) | .129 |  | 0.48 (-0.34 – 1.30) | .250 |  | 0.12 (-0.58 – 0.83) | .733 |  | 0.28 (-0.39 – 0.94) | .416 |  | -0.06 (-0.75 – 0.63) | .863 |  | 0.56 (-0.15 – 1.28) | .120 |  |
| Observations |  | 514 | |  | 514 | |  | 514 | |  | 514 | |  | 514 | |  | 514 | |  | 514 | |  |
| R^2^ / adj. R^2^ |  | .021 / .013 | |  | .012 / .004 | |  | .019 / .012 | |  | .006 / -.002 | |  | .007 / -.001 | |  | .011 / .003 | |  | .016 / .008 | |  |
| F-statistics |  | 2.738* | |  | 1.566 | |  | 2.498* | |  | 0.771 | |  | 0.841 | |  | 1.378 | |  | 2.040 | |  |
| Note: Free will belief scales have been standardized; FW = Free Will subscale; Item = FWI supplementary item; Item key: 1 = Free will is the ability to make different choices even if everything leading up to one’s choice (e.g., the past, the situation, and their desires, beliefs, etc.) were exactly the same; 2 = To be responsible for our present decisions and actions we must also be responsible for all of our prior decisions and actions that led up to the present moment; 3 = Free will is the ability to make a choice based on one’s beliefs and desires such that, if one had different beliefs or desires, one’s choice would have been different as well; 4 = People deserve to be blamed and punished for bad actions only if they acted of their own free will; 5 = People could have free will even if scientists discovered all of the laws that govern all human behavior; 6 = People who harm others deserve to be punished even if punishing them won't produce any positive benefits to either the offender or society—e.g., rehabilitation, deterring other would-be offenders, etc.; 7 = To have free will means that a person’s decisions and actions could not be perfectly predicted by someone else no matter how much information they had | | | | | | | | | | | | | | | | | | | | | | |

| Table 7 (continued)  Moderation models predicting SVO Angle from free will beliefs and supplementary items from the Free Will Inventory | | | | | | | | | | | | | | | | | | | | | | |
| --- | --- | --- | --- | --- | --- | --- | --- | --- | --- | --- | --- | --- | --- | --- | --- | --- | --- | --- | --- | --- | --- | --- |
|  |  |  | 8 | |  | 9 | |  | 10 | |  | 11 | |  | 12 | |  | 13 | |  | 14 | |
|  |  |  | *B (CI)* | *p* |  | *B (CI)* | *p* |  | *B (CI)* | *p* |  | *B (CI)* | *p* |  | *B (CI)* | *p* |  | *B (CI)* | *p* |  | *B (CI)* | *p* |
| (Intercept) |  |  | 27.81 (25.46 – 30.15) | **<.001** |  | 27.63 (25.29 – 29.98) | **<.001** |  | 27.43 (25.02 – 29.85) | **<.001** |  | 27.80 (25.41 – 30.18) | **<.001** |  | 27.26 (24.86 – 29.66) | **<.001** |  | 27.80 (25.42 – 30.19) | **<.001** |  | 27.54 (25.20 – 29.88) | **<.001** |
| Study 3 |  |  | -2.50 (-5.61 – 0.61) | .115 |  | -2.17 (-5.27 – 0.93) | .170 |  | -2.05 (-5.15 – 1.04) | .193 |  | -2.15 (-5.24 – 0.95) | .174 |  | -1.75 (-4.84 – 1.33) | .265 |  | -2.11 (-5.22 – 0.99) | .182 |  | -2.14 (-5.24 – 0.97) | .177 |
| FW |  |  | -0.51 (-1.72 – 0.71) | .411 |  | -0.42 (-1.65 – 0.80) | .498 |  | -0.42 (-1.75 – 0.91) | .533 |  | -0.68 (-1.96 – 0.59) | .293 |  | -1.11 (-2.39 – 0.17) | .089 |  | -0.53 (-1.81 – 0.75) | .415 |  | -0.33 (-1.57 – 0.91) | .600 |
| Item |  |  | 1.28 (0.16 – 2.39) | **.025** |  | 0.41 (-0.48 – 1.30) | .364 |  | -0.10 (-1.34 – 1.15) | .879 |  | 0.75 (-0.46 – 1.96) | .225 |  | 1.84 (0.60 – 3.09) | **.004** |  | -0.19 (-1.29 – 0.91) | .730 |  | -0.33 (-1.14 – 0.48) | .428 |
| FW × Item |  |  | -0.23 (-1.03 – 0.57) | .571 |  | 0.28 (-0.36 – 0.91) | .390 |  | 0.17 (-0.66 – 1.00) | .685 |  | -0.42 (-1.30 – 0.46) | .349 |  | 0.22 (-0.63 – 1.06) | .616 |  | -0.41 (-1.09 – 0.27) | .241 |  | 0.38 (-0.22 – 0.99) | .215 |
| Observations |  |  | 514 | |  | 514 | |  | 514 | |  | 514 | |  | 514 | |  | 514 | |  | 514 | |
| R^2^ / adj. R^2^ |  |  | .015 / .007 | |  | .008 / .000 | |  | .005 / -.003 | |  | .010 / .002 | |  | .021 / .013 | |  | .008 / -.000 | |  | .009 / .001 | |
| F-statistics |  |  | 1.878 | |  | 1.027 | |  | 0.664 | |  | 1.256 | |  | 2.743* | |  | 0.978 | |  | 1.104 | |
| Note: Free will belief scales have been standardized; FW = Free Will subscale; Item = FWI supplementary item; Item key: 8 = People who perform harmful actions ought to be rehabilitated so they no longer pose a threat to society; 9 = If it turned out that people lacked non-physical (or immaterial) souls, then they would lack free will; 10 = People who perform harmful actions ought to be punished so that other potential offenders are deterred from committing similar harmful actions; 11 = To have free will is to be able to cause things to happen in the world without at the same time being caused to make those things happen; 12 = People could be morally responsible even if scientists discovered all of the laws that govern human behavior; 13 = People have free will as long as they are able to do what they want without being coerced or constrained by other people; 14 = If it turned out that people lacked non-physical (or immaterial) souls, then they would lack moral responsibility | | | | | | | | | | | | | | | | | | | | | | |

| Table 8  Moderation models predicting die roll from FAD Free Will, gender, age and income | | | | | | | | | | | | |
| --- | --- | --- | --- | --- | --- | --- | --- | --- | --- | --- | --- | --- |
|  |  | Baseline | |  | Gender | |  | Age | |  | Income | |
|  |  | *B (CI)* | *p* |  | *B (CI)* | *p* |  | *B (CI)* | *p* |  | *B (CI)* | *p* |
| (Intercept) |  | 4.01 (3.80 – 4.21) | **<.001** |  | 4.18 (3.93 – 4.43) | **<.001** |  | 4.50 (4.09 – 4.92) | **<.001** |  | 4.18 (3.85 – 4.52) | **<.001** |
| Study 3 |  | 0.07 (-0.20 – 0.34) | .620 |  | 0.04 (-0.23 – 0.31) | .747 |  | 0.04 (-0.23 – 0.31) | .757 |  | 0.05 (-0.23 – 0.32) | .742 |
| Study 4 |  | 0.01 (-0.29 – 0.31) | .956 |  | -0.03 (-0.33 – 0.27) | .853 |  | -0.07 (-0.37 – 0.23) | .665 |  | -0.01 (-0.31 – 0.29) | .943 |
| FW |  | -0.04 (-0.15 – 0.06) | .419 |  | 0.00 (-0.16 – 0.16) | .987 |  | -0.11 (-0.42 – 0.21) | .507 |  | -0.08 (-0.31 – 0.16) | .518 |
| Female |  |  |  |  | -0.29 (-0.52 – -0.06) | **.013** |  |  |  |  |  |  |
| FW × Female |  |  |  |  | -0.06 (-0.27 – 0.15) | .558 |  |  |  |  |  |  |
| Age |  |  |  |  |  |  |  | -0.01 (-0.02 – -0.00) | **.007** |  |  |  |
| FW × Age |  |  |  |  |  |  |  | 0.00 (-0.01 – 0.01) | .612 |  |  |  |
| Income ($20k - $39k) |  |  |  |  |  |  |  |  |  |  | -0.11 (-0.47 – 0.24) | .530 |
| Income ($40k - $69k) |  |  |  |  |  |  |  |  |  |  | -0.09 (-0.46 – 0.29) | .654 |
| Income ($60k - $79k) |  |  |  |  |  |  |  |  |  |  | -0.35 (-0.76 – 0.06) | .097 |
| Income ($80k+) |  |  |  |  |  |  |  |  |  |  | -0.30 (-0.69 – 0.09) | .130 |
| FW × Income ($20k - $39k) |  |  |  |  |  |  |  |  |  |  | 0.20 (-0.11 – 0.51) | .201 |
| FW × Income ($40k - $69k) |  |  |  |  |  |  |  |  |  |  | -0.13 (-0.45 – 0.19) | .426 |
| FW × Income ($60k - $79k) |  |  |  |  |  |  |  |  |  |  | 0.31 (-0.05 – 0.67) | .088 |
| FW × Income ($80k+) |  |  |  |  |  |  |  |  |  |  | -0.14 (-0.48 – 0.21) | .439 |
| Observations |  | 711 | |  | 711 | |  | 711 | |  | 711 | |
| R^2^ / adj. R^2^ |  | .001 / -.003 | |  | .010 / .003 | |  | .012 / .005 | |  | .022 / .007 | |
| F-statistics |  | 0.304 | |  | 1.485 | |  | 1.684 | |  | 1.423 | |
| Note: Free will belief scales have been standardized; FW = FAD Free Will subscale | | | | | | | | | | | | |

| Table 9  Moderation models predicting die roll from FAD Free Will, education, non-naïveté, and religion | | | | | | | | | |
| --- | --- | --- | --- | --- | --- | --- | --- | --- | --- |
|  |  | Education | |  | Prev. FW | |  | Religion | |
|  |  | *B (CI)* | *p* |  | *B (CI)* | *p* |  | *B (CI)* | *p* |
| (Intercept) |  | 3.99 (3.78 – 4.20) | **<.001** |  | 4.11 (3.89 – 4.33) | **<.001** |  | 4.25 (4.00 – 4.51) | **<.001** |
| Study 3 |  | 0.07 (-0.20 – 0.34) | .602 |  | 0.08 (-0.19 – 0.35) | .585 |  | 0.02 (-0.25 – 0.30) | .861 |
| Study 4 |  | 0.02 (-0.28 – 0.31) | .917 |  | -0.00 (-0.30 – 0.30) | .999 |  | -0.02 (-0.32 – 0.27) | .890 |
| FW |  | -0.06 (-0.17 – 0.05) | .293 |  | -0.03 (-0.16 – 0.09) | .627 |  | -0.08 (-0.25 – 0.09) | .332 |
| HS |  | 0.09 (-0.25 – 0.42) | .622 |  |  |  |  |  |  |
| FW × HS |  | 0.10 (-0.19 – 0.39) | .498 |  |  |  |  |  |  |
| Prev. FW |  |  |  |  | -0.30 (-0.54 – -0.06) | **.014** |  |  |  |
| FW × Prev. FW |  |  |  |  | -0.06 (-0.28 – 0.17) | .626 |  |  |  |
| Christian |  |  |  |  |  |  |  | -0.44 (-0.69 – -0.18) | **<.001** |
| Other rel. |  |  |  |  |  |  |  | -0.30 (-0.72 – 0.11) | .152 |
| Rel. Str. |  |  |  |  |  |  |  | -0.02 (-0.07 – 0.04) | .551 |
| FW × Christian |  |  |  |  |  |  |  | 0.12 (-0.12 – 0.37) | .331 |
| FW × Other rel. |  |  |  |  |  |  |  | 0.09 (-0.24 – 0.42) | .583 |
| FW × Rel. Str. |  |  |  |  |  |  |  | -0.04 (-0.09 – 0.01) | .096 |
| Christian × Rel. Str. |  |  |  |  |  |  |  | 0.01 (-0.07 – 0.10) | .757 |
| Other × Rel. Str. |  |  |  |  |  |  |  | -0.14 (-0.29 – 0.00) | .058 |
| FW × Christian × Rel. Str. |  |  |  |  |  |  |  | 0.06 (-0.02 – 0.14) | .156 |
| FW × Other × Rel. Str. |  |  |  |  |  |  |  | 0.14 (0.03 – 0.26) | **.016** |
| Observations |  | 711 | |  | 711 | |  | 705 | |
| R^2^ / adj. R^2^ |  | .002 / -.005 | |  | .010 / .003 | |  | .040 / .022 | |
| F-statistics |  | 0.350 | |  | 1.437 | |  | 2.213** | |
| Note: Free will belief scales have been standardized; FW = FAD Free Will subscale; HS = High-school is highest level of education completed; Prev. FW = Previous experience with free will research; Rel. Str. = strength of identification with one’s religious group (standardized) | | | | | | | | | |

| Table 10  Moderation models predicting die roll from moral identity and social desirability | | | | | | | | | |
| --- | --- | --- | --- | --- | --- | --- | --- | --- | --- |
|  |  | Internalization | |  | Symbolization | |  | Social Desirability | |
|  |  | *B (CI)* | *p* |  | *B (CI)* | *p* |  | *B (CI)* | *p* |
| (Intercept) |  | 4.03 (3.82 – 4.24) | **<.001** |  | 3.99 (3.79 – 4.20) | **<.001** |  | 3.99 (3.78 – 4.20) | **<.001** |
| Study 3 |  | 0.05 (-0.22 – 0.32) | .704 |  | 0.08 (-0.19 – 0.35) | .556 |  | 0.08 (-0.19 – 0.35) | .576 |
| Study 4 |  | -0.07 (-0.36 – 0.23) | .667 |  | 0.03 (-0.27 – 0.33) | .852 |  |  |  |
| FW |  | 0.01 (-0.09 – 0.12) | .817 |  | -0.03 (-0.13 – 0.08) | .626 |  | -0.07 (-0.20 – 0.05) | .256 |
| Internalization |  | -0.23 (-0.35 – -0.12) | **<.001** |  |  |  |  |  |  |
| FW × Internalization |  | 0.01 (-0.09 – 0.10) | .889 |  |  |  |  |  |  |
| Symbolization |  |  |  |  | -0.07 (-0.16 – 0.01) | .092 |  |  |  |
| FW × Symbolization |  |  |  |  | 0.01 (-0.06 – 0.09) | .768 |  |  |  |
| Soc. Des |  |  |  |  |  |  |  | 0.01 (-0.04 – 0.06) | .708 |
| FW × Soc. Des |  |  |  |  |  |  |  | 0.02 (-0.02 – 0.07) | .312 |
| Observations |  | 711 | |  | 711 | |  | 514 | |
| R^2^ / adj. R^2^ |  | .025 / .018 | |  | .005 / -.002 | |  | .005 / -.003 | |
| F-statistics |  | 3.554** | |  | 0.752 | |  | 0.650 | |
| Note: Free will belief, moral identity, and social desirability scales have been standardized; FW = FAD Free Will subscale; Soc. Des. = Social Desirability | | | | | | | | | |

| Table 11  Moderation models predicting die roll from free will beliefs as measured by the FAD Plus | | | | | | | | | | | | |
| --- | --- | --- | --- | --- | --- | --- | --- | --- | --- | --- | --- | --- |
|  |  | Baseline | |  | Scientific Determinism | |  | Fatalistic Determinism | |  | Unpredictability | |
|  |  | *B (CI)* | *p* |  | *B (CI)* | *p* |  | *B (CI)* | *p* |  | *B (CI)* | *p* |
| (Intercept) |  | 4.00 (3.80 – 4.21) | **<.001** |  | 4.00 (3.80 – 4.20) | **<.001** |  | 4.00 (3.79 – 4.20) | **<.001** |  | 4.01 (3.80 – 4.21) | **<.001** |
| Study 3 |  | 0.07 (-0.20 – 0.34) | .601 |  | 0.07 (-0.20 – 0.34) | .596 |  | 0.07 (-0.20 – 0.34) | .589 |  | 0.07 (-0.21 – 0.34) | .636 |
| FW |  | -0.07 (-0.19 – 0.06) | .284 |  | -0.07 (-0.20 – 0.05) | .236 |  | -0.07 (-0.20 – 0.05) | .248 |  | -0.06 (-0.19 – 0.06) | .306 |
| SD |  |  |  |  | 0.04 (-0.10 – 0.17) | .569 |  |  |  |  |  |  |
| FW × SD |  |  |  |  | -0.07 (-0.19 – 0.04) | .201 |  |  |  |  |  |  |
| FD |  |  |  |  |  |  |  | -0.03 (-0.14 – 0.07) | .547 |  |  |  |
| FW × FD |  |  |  |  |  |  |  | -0.02 (-0.11 – 0.08) | .730 |  |  |  |
| Unpred. |  |  |  |  |  |  |  |  |  |  | 0.03 (-0.11 – 0.17) | .663 |
| FW × Unpred. |  |  |  |  |  |  |  |  |  |  | -0.01 (-0.13 – 0.11) | .836 |
| Observations |  | 514 | |  | 514 | |  | 514 | |  | 514 | |
| R^2^ / adj. R^2^ |  | .003 / -.001 | |  | .006 / -.002 | |  | .004 / -.004 | |  | .003 / -.005 | |
| F-statistics |  | 0.680 | |  | 0.773 | |  | 0.492 | |  | 0.394 | |
| Note: Free will belief scales have been standardized; FW = Free Will subscale; SD = Scientific Determinism subscale; FD = Fatalistic Determinism subscale; Unpred = Unpredictability subscale | | | | | | | | | | | | |

| Table 12  Moderation models predicting die roll from free will beliefs as measured by the Free Will Inventory | | | | | | | | | |
| --- | --- | --- | --- | --- | --- | --- | --- | --- | --- |
|  |  | Baseline | |  | Determinism | |  | Dualism | |
|  |  | *B (CI)* | *p* |  | *B (CI)* | *p* |  | *B (CI)* | *p* |
| (Intercept) |  | 4.01 (3.80 – 4.21) | **<.001** |  | 3.99 (3.79 – 4.20) | **<.001** |  | 4.00 (3.80 – 4.21) | **<.001** |
| Study 3 |  | 0.07 (-0.20 – 0.34) | .623 |  | 0.08 (-0.19 – 0.35) | .572 |  | 0.05 (-0.22 – 0.32) | .699 |
| FW |  | -0.06 (-0.17 – 0.04) | .244 |  | -0.06 (-0.17 – 0.04) | .244 |  | -0.02 (-0.13 – 0.09) | .737 |
| Determinism |  |  |  |  | 0.01 (-0.10 – 0.12) | .874 |  |  |  |
| FW × Determinism |  |  |  |  | -0.02 (-0.10 – 0.05) | .545 |  |  |  |
| Dualism |  |  |  |  |  |  |  | -0.11 (-0.20 – -0.03) | **.009** |
| FW × Dualism |  |  |  |  |  |  |  | 0.02 (-0.03 – 0.07) | .481 |
| Observations |  | 514 | |  | 514 | |  | 514 | |
| R^2^ / adj. R^2^ |  | .003 / -.001 | |  | .004 / -.004 | |  | .017 / .010 | |
| F-statistics |  | 0.784 | |  | 0.488 | |  | 2.249 | |
| Note: Free will belief scales have been standardized; FW = Free Will subscale | | | | | | | | | |

| Table 13  Moderation models predicting die roll from free will beliefs and supplementary items from the Free Will Inventory | | | | | | | | | | | | | | | | | | | | | | |
| --- | --- | --- | --- | --- | --- | --- | --- | --- | --- | --- | --- | --- | --- | --- | --- | --- | --- | --- | --- | --- | --- | --- |
|  |  | 1 | |  | 2 | |  | 3 | |  | 4 | |  | 5 | |  | 6 | |  | 7 | |  |
|  |  | *B (CI)* | *p* |  | *B (CI)* | *p* |  | *B (CI)* | *p* |  | *B (CI)* | *p* |  | *B (CI)* | *p* |  | *B (CI)* | *p* |  | *B (CI)* | *p* |  |
| (Intercept) |  | 3.98 (3.77 – 4.20) | **<.001** |  | 4.01 (3.80 – 4.22) | **<.001** |  | 4.01 (3.80 – 4.22) | **<.001** |  | 4.01 (3.81 – 4.22) | **<.001** |  | 3.97 (3.75 – 4.18) | **<.001** |  | 4.00 (3.79 – 4.21) | **<.001** |  | 3.99 (3.78 – 4.20) | **<.001** |  |
| Study 3 |  | 0.06 (-0.21 – 0.33) | .668 |  | 0.07 (-0.20 – 0.34) | .617 |  | 0.07 (-0.20 – 0.34) | .628 |  | 0.08 (-0.19 – 0.35) | .580 |  | 0.08 (-0.19 – 0.35) | .549 |  | 0.06 (-0.20 – 0.33) | .637 |  | 0.06 (-0.21 – 0.33) | .650 |  |
| FW |  | -0.03 (-0.15 – 0.08) | .548 |  | -0.07 (-0.18 – 0.05) | .249 |  | -0.02 (-0.14 – 0.09) | .729 |  | -0.07 (-0.18 – 0.05) | .251 |  | -0.07 (-0.19 – 0.06) | .285 |  | -0.07 (-0.18 – 0.05) | .254 |  | -0.04 (-0.16 – 0.07) | .469 |  |
| Item |  | -0.08 (-0.20 – 0.04) | .172 |  | 0.01 (-0.09 – 0.11) | .835 |  | -0.10 (-0.22 – 0.01) | .067 |  | -0.02 (-0.11 – 0.07) | .643 |  | 0.03 (-0.08 – 0.13) | .585 |  | 0.02 (-0.07 – 0.11) | .689 |  | -0.03 (-0.13 – 0.06) | .476 |  |
| FW × Item |  | 0.05 (-0.03 – 0.13) | .246 |  | -0.01 (-0.08 – 0.06) | .809 |  | 0.00 (-0.07 – 0.07) | .980 |  | -0.03 (-0.09 – 0.03) | .375 |  | 0.03 (-0.03 – 0.09) | .294 |  | 0.01 (-0.05 – 0.07) | .715 |  | 0.02 (-0.04 – 0.09) | .463 |  |
| Observations |  | 514 | |  | 514 | |  | 514 | |  | 514 | |  | 514 | |  | 514 | |  | 514 | |  |
| R^2^ / adj. R^2^ |  | .010 / .003 | |  | .003 / -.005 | |  | .010 / .002 | |  | .005 / -.002 | |  | .006 / -.002 | |  | .004 / -.004 | |  | .005 / -.003 | |  |
| F-statistics |  | 1.344 | |  | 0.418 | |  | 1.255 | |  | 0.680 | |  | 0.707 | |  | 0.463 | |  | 0.675 | |  |
| Note: Free will belief scales have been standardized; FW = Free Will subscale; Item = FWI supplementary item; Item key: 1 = Free will is the ability to make different choices even if everything leading up to one’s choice (e.g., the past, the situation, and their desires, beliefs, etc.) were exactly the same; 2 = To be responsible for our present decisions and actions we must also be responsible for all of our prior decisions and actions that led up to the present moment; 3 = Free will is the ability to make a choice based on one’s beliefs and desires such that, if one had different beliefs or desires, one’s choice would have been different as well; 4 = People deserve to be blamed and punished for bad actions only if they acted of their own free will; 5 = People could have free will even if scientists discovered all of the laws that govern all human behavior; 6 = People who harm others deserve to be punished even if punishing them won't produce any positive benefits to either the offender or society—e.g., rehabilitation, deterring other would-be offenders, etc.; 7 = To have free will means that a person’s decisions and actions could not be perfectly predicted by someone else no matter how much information they had | | | | | | | | | | | | | | | | | | | | | | |

| Table 13 (continued)  Moderation models predicting die roll from free will beliefs and supplementary items from the Free Will Inventory | | | | | | | | | | | | | | | | | | | | | | |
| --- | --- | --- | --- | --- | --- | --- | --- | --- | --- | --- | --- | --- | --- | --- | --- | --- | --- | --- | --- | --- | --- | --- |
|  |  |  | 8 | |  | 9 | |  | 10 | |  | 11 | |  | 12 | |  | 13 | |  | 14 | |
|  |  |  | *B (CI)* | *p* |  | *B (CI)* | *p* |  | *B (CI)* | *p* |  | *B (CI)* | *p* |  | *B (CI)* | *p* |  | *B (CI)* | *p* |  | *B (CI)* | *p* |
| (Intercept) |  |  | 4.00 (3.80 – 4.21) | **<.001** |  | 4.00 (3.80 – 4.20) | **<.001** |  | 3.99 (3.78 – 4.20) | **<.001** |  | 4.01 (3.80 – 4.21) | **<.001** |  | 3.95 (3.74 – 4.16) | **<.001** |  | 4.00 (3.79 – 4.20) | **<.001** |  | 4.00 (3.80 – 4.20) | **<.001** |
| Study 3 |  |  | 0.08 (-0.20 – 0.35) | .582 |  | 0.08 (-0.20 – 0.35) | .585 |  | 0.07 (-0.20 – 0.34) | .616 |  | 0.07 (-0.20 – 0.34) | .630 |  | 0.10 (-0.17 – 0.37) | .486 |  | 0.08 (-0.19 – 0.35) | .549 |  | 0.09 (-0.18 – 0.36) | .519 |
| FW |  |  | -0.06 (-0.17 – 0.04) | .249 |  | -0.07 (-0.17 – 0.04) | .220 |  | -0.04 (-0.15 – 0.08) | .506 |  | -0.07 (-0.18 – 0.04) | .199 |  | -0.07 (-0.18 – 0.04) | .199 |  | -0.04 (-0.15 – 0.07) | .487 |  | -0.08 (-0.19 – 0.03) | .155 |
| Item |  |  | -0.05 (-0.15 – 0.05) | .326 |  | 0.01 (-0.07 – 0.09) | .806 |  | -0.04 (-0.15 – 0.07) | .457 |  | 0.03 (-0.07 – 0.14) | .546 |  | 0.05 (-0.06 – 0.15) | .411 |  | -0.07 (-0.16 – 0.03) | .168 |  | -0.02 (-0.09 – 0.05) | .546 |
| FW × Item |  |  | 0.04 (-0.02 – 0.11) | .206 |  | -0.02 (-0.07 – 0.04) | .512 |  | 0.03 (-0.05 – 0.10) | .481 |  | 0.00 (-0.07 – 0.08) | .938 |  | 0.08 (0.01 – 0.15) | **.036** |  | 0.00 (-0.06 – 0.06) | .920 |  | -0.05 (-0.10 – 0.01) | .083 |
| Observations |  |  | 514 | |  | 514 | |  | 514 | |  | 514 | |  | 514 | |  | 514 | |  | 514 | |
| R^2^ / adj. R^2^ |  |  | .007 / -.001 | |  | .004 / -.004 | |  | .006 / -.002 | |  | .004 / -.004 | |  | .012 / .004 | |  | .007 / -.001 | |  | .010 / .002 | |
| F-statistics |  |  | 0.925 | |  | 0.509 | |  | 0.712 | |  | 0.482 | |  | 1.576 | |  | 0.876 | |  | 1.311 | |
| Note: Free will belief scales have been standardized; FW = Free Will subscale; Item = FWI supplementary item; Item key: 8 = People who perform harmful actions ought to be rehabilitated so they no longer pose a threat to society; 9 = If it turned out that people lacked non-physical (or immaterial) souls, then they would lack free will; 10 = People who perform harmful actions ought to be punished so that other potential offenders are deterred from committing similar harmful actions; 11 = To have free will is to be able to cause things to happen in the world without at the same time being caused to make those things happen; 12 = People could be morally responsible even if scientists discovered all of the laws that govern human behavior; 13 = People have free will as long as they are able to do what they want without being coerced or constrained by other people; 14 = If it turned out that people lacked non-physical (or immaterial) souls, then they would lack moral responsibility | | | | | | | | | | | | | | | | | | | | | | |

1. It is important to note that there are several ways in which our manipulation differs to that used in Study 2 of Vohs and Schooler (2008). They (1) used 15 statements instead of 10, (2) had participants just read but not re-write statements, and (3) conducted their manipulation in person rather than online. Although our procedure is overall quite similar, it is possible that the lack of success in our manipulation is attributable to these differences (Schooler et al., 2014). We discuss this matter further below. [↑](#footnote-ref-1)
2. Note, however, that we only asked whether people had participated in research on free will *in general*, rather than asking about previous experience with any particular method or measurement. [↑](#footnote-ref-2)
3. For many models, we observed significant parameter estimates with non-significant overall model F tests. Interested readers will find these in the regression tables, but we refrain from interpreting these findings, instead treating them as non-significant effects. [↑](#footnote-ref-3)
4. Following the recommendation of Galen (2012), we tested an additional model with a quadratic term for strength of religious identification. This model did not offer a significant improvement over the model without the quadratic term, so we omit this model for brevity. [↑](#footnote-ref-4)
5. Additionally, we observed a positive three-way interaction between FWB, Other Religion, and strength of religious identification such that, compared to non-religious participants with average FWB, and average identification with their (non-)religious group, participants were more likely to cheat if they were (1) religious but not Christian, (2) strongly identified with their religious group, and (3) have a strong belief in free will. However, because this model is based on only 71 participants identifying as religious but not Christian, we are especially hesitant to read into this complex pattern. [↑](#footnote-ref-5)
6. These items were worded as follows. Item 1: “Free will is the ability to make different choices even if everything leading up to one’s choice (e.g., the past, the situation, and their desires, beliefs, etc.) were exactly the same”; Item 3: “Free will is the ability to make a choice based on one’s beliefs and desires such that, if one had different beliefs or desires, one’s choice would have been different as well”; and finally Item 12 “People could be morally responsible even if scientists discovered all of the laws that govern human behavior.” [↑](#footnote-ref-6)
7. One possibility is that, in an online setting, forcing participants to stay on the page for each sentence for 30 seconds before they could progress led them to distraction. [↑](#footnote-ref-7)
8. This effect is less than one fifth of the original effect size reported by Vohs & Schooler (2008) which decreased FWBs by more than 1 SD, using an earlier version of the FAD-Plus Free Will subscale as a manipulation check measure. At the time of our Study 4, the Vohs and Schooler study was to our knowledge the most methodologically similar study to our own, and so was used to determine our sample size. [↑](#footnote-ref-8)
9. It should also be noted that Monroe et al. (2016) also included a single item manipulation check (probing agreement with the statement “I have free will”) which produced a larger effect size (*d* = .64) than their FWI-based manipulation check. This suggests that – for reasons that are not exactly clear – the specific manipulation check measure used is also an important design consideration, even if they appear to be measuring identical constructs. [↑](#footnote-ref-9)
10. Note that a non-significant result would not necessarily preclude cheating. A whole-sample KS-test could return null results in situations where some people systematically over-report and others under-report, such that they cancel out each other’s effect on the overall distribution. [↑](#footnote-ref-10)
